# Revascularization to the bone tunnel wall after anterior cruciate ligament reconstruction may relate to the distance from the vessels

**DOI:** 10.1186/s43019-020-00070-3

**Published:** 2020-10-06

**Authors:** Yuji Arai, Kunio Hara, Hiroaki Inoue, Hitoshi Kanamura, Shuji Nakagawa, Satoru Atsumi, Yasuo Mikami

**Affiliations:** 1 Department of Sports and Para-Sports Medicine, Graduate School of Medical Science, Kyoto Prefectural University of Medicine, 465, Kajiicho, Kawaramachi-Hirokoji, Kamigyo-Ku, Kyoto, Kyoto 602-8566 Japan; 2 Japan Community Health care Organization Kyoto Kuramaguchi Medical Center, 27, Shimofusacho, Koyama, Kita-Ku, Kyoto, Kyoto 603-8151 Japan; 3 Department of Orthopaedics, Graduate School of Medical Science, Kyoto Prefectural University of Medicine, 465, Kajiicho, Kawaramachi-Hirokoji, Kamigyo-Ku, Kyoto, Kyoto 602-8566 Japan; 4 Department of Rehabilitation Medicine, Graduate School of Medical Science, Kyoto Prefectural University of Medicine, 465, Kajiicho, Kawaramachi-Hirokoji, Kamigyo-Ku, Kyoto, Kyoto 602-8566 Japan

**Keywords:** Anterior cruciate ligament, Bone tunnel wall, Magnetic resonance angiography, Revascularization

## Abstract

**Purpose:**

We use magnetic resonance angiography to evaluate the difference of vascular ingrowth to the bone tunnel on the anterior and posterior walls quantitatively after anterior cruciate ligament reconstruction.

**Materials and methods:**

One hundred patients underwent anterior cruciate ligament reconstruction with multi-stranded semitendinosus tendons. They were retrospectively divided into those who underwent magnetic resonance angiography 2, 3, 4 to 6, and ≥ 7 months after surgery. The mean signal-to-noise ratios of the bone tunnel walls in the femur and tibia from the digital data were measured and compared for the anterior and posterior walls.

**Results:**

The signal-to-noise ratio of the posterior wall of the femoral bone tunnel was significantly higher than that of the anterior wall in each group. On the tibial side, the signal-to-noise ratio of the anterior wall was significantly higher than that of the posterior wall at ≥4 months after surgery.

**Conclusions:**

This study showed that the blood flow after anterior cruciate ligament reconstruction to the femoral bone tunnel is maintained from the posterior wall, and is maintained to the tibial side from the anterior wall 4 months postoperatively. Revascularization to the bone tunnel wall after anterior cruciate ligament reconstruction may relate to the distance from the vessels.

## Introduction

The anterior cruciate ligament (ACL) is a support mechanism responsible for stabilization of the knee joint. Recovery of ACL function after injury is needed because decline of knee joint function occurs with ACL injury and has a significant influence on both daily and sports activities. As surgical therapy, ACL reconstruction is widely performed, in which the graft is passed through bone tunnels made in the tibia and femur. For the tendon graft material, the hamstring, bone-patellar tendon, iliotibial tract, quadriceps femoris tendon, and allograft tissue are used [1–4]. A hamstring autograft is one of the most frequently used grafts and has shown favorable clinical results [4–9]. However, the ability to engage in sports activities may require a long time to return [10].

Remodeling processes, such as remodeling of the tendon graft and consolidation of the bone-tendon junction, are important as biological factors that determine the treatment outcome of ACL reconstruction when the hamstring tendon is used as a graft [11, 12]. Although tendon grafts tend to necrose immediately after surgery, the remodeling processes occur with restarting of the blood flow [13]. Excessive loading may cause failure if the blood flow is insufficient, because the blood flow after ACL reconstruction is important [14, 15]. Analysis of the blood flow in vivo is useful for the elucidation of the remodeling process [16]. In previous studies, reinitiation of blood flow to the bone tunnel wall has been shown to be involved in the remodeling process [17] and is supplied from the posterior middle genicular artery to the femoral bone tunnel, and from the anterior inferior genicular artery to the tibial side [18]. Even in the same bone tunnel hole, reinitiation of blood flow to the bone tunnel wall may differ for the anterior and posterior walls. However, the uniformity of revascularization to the bone tunnel wall is still unclear. Elucidating the differentiation of revascularization may provide useful information on the relationship between the remodeling process and the location of the bone tunnel.

From this background, we hypothesized that the blood supply is biased after ACL reconstruction. The purpose of this study was to quantitatively assess the hemodynamics to the bone tunnel on the anterior and posterior walls over time after ACL reconstruction.

## Materials and methods

### Subjects

**Fig. 1:**
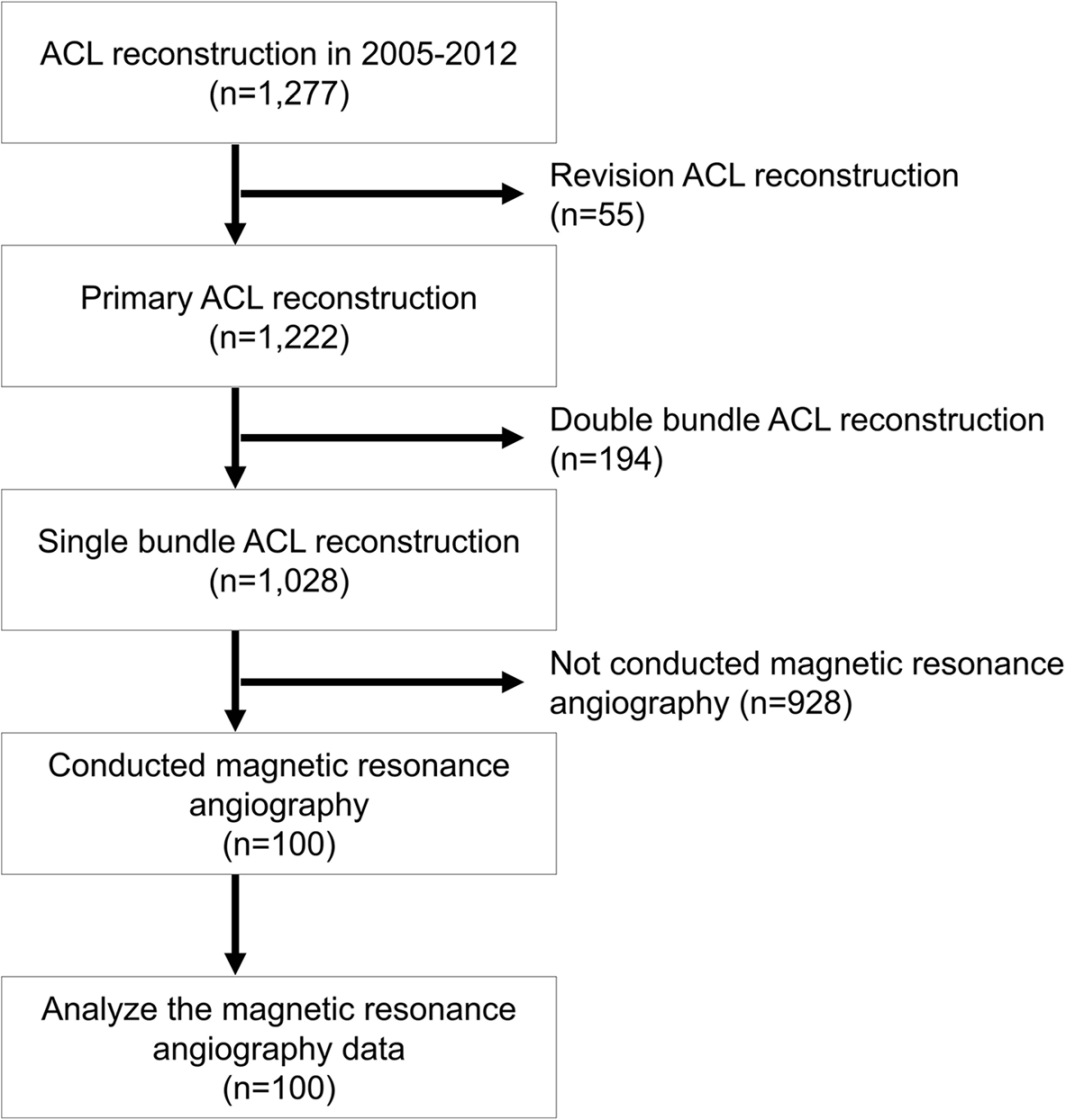
The flowchart of patient selection

From 2005 to 2012, 1277 patients received ACL reconstruction in our hospital. We excluded 55 patients who underwent revision surgery and 194 patients who had double-bundle reconstruction. No patients received other ligament reconstruction simultaneously. Of the remaining 1028 patients, 100 patients who consented to this study were tested with magnetic resonance angiography (MRA) (Fig. 1). Therefore, the study population consisted of 100 patients (100 knees; 36 men and 64 women) who underwent single-bundle ACL reconstruction using four-stranded hamstring tendons. The mean age of the patients was 23.7 years (range 14–47 years). Table 1 lists the patient demographic data. The inclusion criteria were single-bundle reconstruction with multi-stranded hamstring and initial injury. The exclusion criteria were double-bundle reconstruction, bone-tendon-bone graft, revision, and complex ligament injury. Ethical approval for this study was obtained from the Ethical Review Board of our hospital (ERB-C-159-1). All procedures were in accordance with the ethical standards of our institute’s Ethical Review Board and the Helsinki Declaration of 1975, as revised in 2000.

**Table 1:** Patient demographic data

	Cases (male/female)	Mean age (range)
Group A	46 (18/28)	23.6 (15–47)
Group B	17 (3/14)	24.1 (16–35)
Group C	16 (10/6)	25.3 (15–38)
Group D	21 (6/15)	22.2 (14–44)

### Surgical technique

All reconstructive procedures were performed using the inside-out technique for the tibial tunnel and the trans-tibial technique for the femoral tunnel under arthroscopic observation. The semitendinosus tendon was harvested and arranged into a fourfold loop with a diameter of 7–9 mm in all cases. One end of the stump was sutured with 0 Surgilon thread (Kendall, Hampshire, UK), and four sutures were placed for fixation. The Endobutton CL Fixation System (Smith & Nephew, Andover, MA, USA) was attached to the looped end of the graft for femoral fixation. An artificial ligament (Telos, Tokyo, Japan) made of polyester was connected to the other sutured end of the graft for tibial fixation. A tibial tunnel, 8 or 9 mm in diameter for men and 7 or 8 mm for women, was drilled. Subsequently, a femoral tunnel, the same diameter and 25 mm in length, was drilled using the trans-tibial technique.

Nylon threads attached to the Endobutton CL-semitendinosus tendon complex were connected to the end of the passing pin and inserted into the tunnel. The ceramic button was inserted into the tibial tunnel. The Telos artificial ligament with the semitendinosus tendon was ligated through the hole of the ceramic button in a 20° flexion position with maximum manual pulling. The grafts in the bone tunnels of the femoral and tibial sides were 10–15 mm in length. After insertion of the graft, we confirmed the absence of roof impingement.

### Postoperative management

The knee was fixed with a splint (Light Splint; ALCARE, Tokyo, Japan) in the 20° flexion position. Knee range-of-motion exercises were begun on the second day after surgery. Partial weight bearing was initiated 7 days after surgery. At 4 weeks postoperatively, walking was permitted. However, some patients with repair of the meniscus were restricted to knee flexion under 90° for 2 weeks after surgery and under 120° for 4 weeks after surgery. They were also instructed to delay their weight-bearing protocol for a week. Jogging was encouraged 8 weeks after reconstruction in all patients. Return to sports was permitted 6 months after surgery, following evaluation of muscle strength.

### MRA

All patients underwent MRA after ACL reconstruction. The patients were retrospectively divided into those undergoing MRA at 2 (group A), 3 (group B), 4 to 6 (group C), and ≥ 7 months (group D) after ACL reconstruction (46, 17, 16, and 21 patients, respectively) in accordance with a previous report [19]. The patients in group D underwent MRA on average in 9.8 months (from 7 to 24 months). The timing of image acquisition was set in consideration of the postoperative rehabilitation protocol. As return to playing sports was permitted 6 months after surgery, images were acquired at 2, 3, and 4 to 6 months during medical and athletic rehabilitation. As remodeling continues beyond 7 months after surgery, clinical evaluation was performed for up to 2 years after surgery. MRA was performed after 4 months in patients treated between August 2005 and March 2008, and up to 3 months after surgery in those treated between April 2008 and December 2012. Imaging was performed using a 1.5-T magnetic resonance imager (Gyroscan; Philips Medical Systems, Best, The Netherlands) with a knee coil, as described previously [18, 20]. For fat suppression images, the water-selective excitation technique was used. Contrast-enhanced MRA was performed after intravenous injection of gadolinium-diethylenetriamine pentaacetic acid (Gd-DTPA; 0.1 mmol/kg body weight). To reduce the background tissue signal, a mask image was obtained immediately after administration of the contrast agent. The imaging parameters were as follows: repetition time (TR) = 13 ms, echo time (TE) = 5.2 ms, flip angle (FA) = 25°, field of view = 150 mm, slice thickness = 6 mm, gap between slices = 3 mm, with a 224 × 180 matrix. Digital subtraction angiography was performed every 24 s. Imaging was confined to oblique and sagittal sections to allow for alignment of the popliteal artery. The total imaging time was 140 s. In the first step of reconstruction, the mask image data set was subtracted from the contrast-enhanced data set.

### Analysis of MRA data

**Fig. 2:**
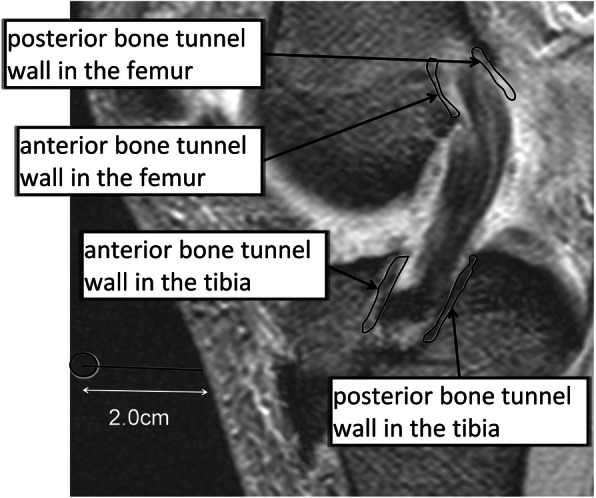
The setting method of the region of interest is indicated. Slices in which the tendon graft and bone tunnel walls were most linear in the longitudinal direction in an oblique sagittal plane were used. On the femoral side, the inner wall of the bone tunnel, 5 mm anterior and posterior to the center of the tendon graft on Blumensaat’s line and parallel to this line, was regarded as the bone tunnel wall, which was manually enclosed as a linear region with a width of 2 mm. On the tibial side, the inner wall of the bone tunnel, 5 mm anterior and posterior to the center of the tendon graft at the bone tunnel opening on the joint surface side parallel to the joint surface, was regarded as the bone tunnel wall, which was enclosed in the same way as the femur side. The measured signal intensity was divided by the signal intensity of air, 2.0 cm anterior to the tibial tuberosity, in order to obtain the signal-to-noise ratio as normalization of signal intensity

MRA DICOM images were quantified using image processing software (OsiriX®, Pixmeo, Geneva, Switzerland). To set the region of interest (ROI), slices in which the tendon graft and bone tunnel walls were most linear in the longitudinal direction in an oblique sagittal plane were used. On the femoral side, the inner wall of the bone tunnel, 5 mm anterior and posterior to the center of the tendon graft on Blumensaat’s line and parallel to this line, was regarded as the bone tunnel wall, which was manually enclosed as a linear region with a width of 2 mm. On the tibial side, the inner wall of the bone tunnel, 5 mm anterior and posterior to the center of the tendon graft at the bone tunnel opening on the joint surface side parallel to the joint surface, was regarded as the bone tunnel wall, and the wall was manually enclosed as a linear region with a width of 2 mm. The signal intensity was measured independently by two different orthopedic surgeons in a blinded manner, and each measurement was repeated twice, the interval was 1 month, with the average value regarded as the results. The measured signal intensity was divided by the signal intensity of air, 2.0 cm anterior to the tibial tuberosity, in order to obtain the signal-to-noise ratio (SNR), and the signal intensity was normalized [19] (Fig. 2). It was defined as follows:

SNR = (signal intensity of ROI)/(signal intensity of air).

### Statistical analysis

Measurements were expressed as the means ± standard deviation. Two-way analysis of variance (ANOVA) was used to analyze the mean SNR with group (anterior and posterior) and postoperative period (2, 3, 4–6, ≥ 7 months) as between-subject factors; Tukey’s post hoc test was performed for multiple comparisons. Intraclass correlation coefficients (ICCs) were calculated with a two-way random model. Statistical analyses were conducted using SPSS (version 21.0 for Windows; IBM, Chicago, IL). In all analyses, *p* < 0.05 was taken to indicate statistical significance. We also investigated return to sports and re-injury.

## Results

The results concerning interobserver agreement for the SNR and intraobserver reliability of the two observers are 0.971 (95% confidence interval [CI] = 0.928–0.988), 0.995 (95% CI = 0.987–0.998), and 0.980 (95% CI = 0.950–0.992), respectively.

Concurrent injuries are shown in Table 2. The medial meniscus of six, one, and three patients was repaired simultaneously in groups A, B, and C, respectively. Regarding the findings of cartilage, six cases were grade 1 of the International Cartilage Repair Society, and one case of group D was grade 4 (Table 2). In all cases there was no surgery performed for cartilage lesions.

**Table 2:** Concurrent injuries

	Meniscal injury		Cartilage injury
	Lateral	Medial	
Group A	13	13	2
Group B	3	4	0
Group C	5	5	1
Group D	4	2	3

**Fig. 3:**
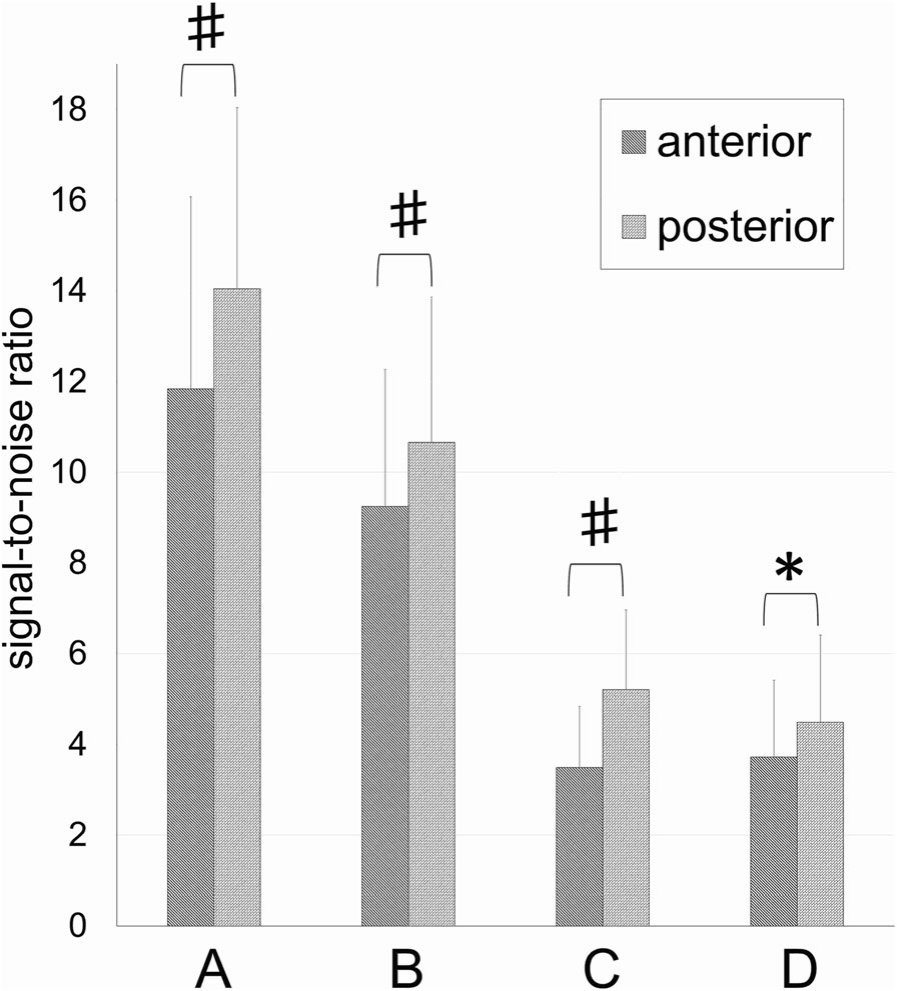
Signal-to-noise ratio of the anterior and posterior bone tunnel walls in the femur. *Error bars* indicate the standard deviation. * *p* < 0.01, # *p* < 0.001

In the comparison between the anterior and posterior side of the bone tunnel wall, the average SNR of the posterior wall of the femoral bone tunnel in group A was 14.0 **±** 4.00, which was significantly higher than that of the anterior wall, which was 11.8 **±** 4.23 (*p* < 0.001) (Fig. 3). The average SNRs in groups B, C, and D were 9.26 ± 3.01, 3.49 ± 1.34, and 3.73 ± 1.68 on the anterior wall, and 10.7 ± 3.20, 5.21 ± 1.76, and 4.49 ± 1.92 on the posterior wall, respectively. The SNRs in groups B, C, and D were lower in both the anterior and posterior walls compared with group A (*p* = 0.03, *p* < 0.001, *p* < 0.001 in anterior, *p* = 0.002, *p* < 0.001, *p* < 0.001 in posterior, respectively). In the comparison of the anterior and posterior walls in each group, the SNR was higher in the posterior wall compared with the anterior wall, similar to group A (*p* = 0.009, *p* < 0.001, *p* = 0.003, respectively).

**Fig. 4:**
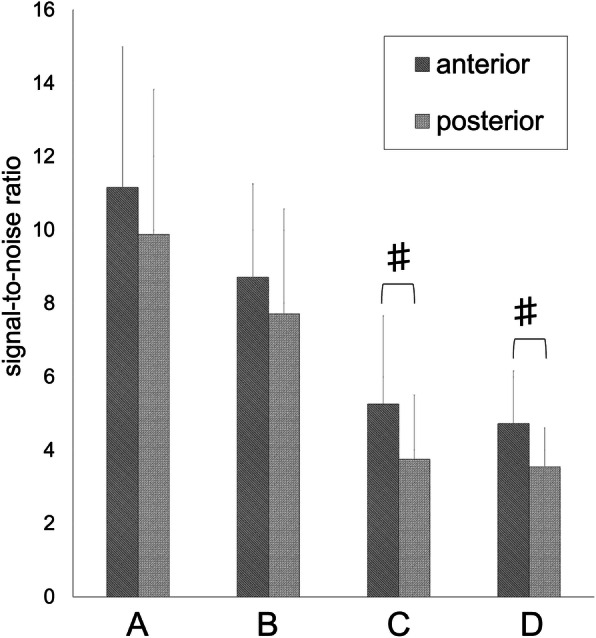
Signal-to-noise ratio of the anterior and posterior bone tunnel walls in the tibia. *Error bars* indicate the standard deviation. # *p* < 0.001

On the tibial side, the SNRs in groups C and D were 5.25 ± 2.41 and 4.72 ± 1.44 on the anterior wall, and 3.75 ± 1.75 and 3.54 ± 1.06 on the posterior wall, respectively (Fig. 4). The average SNR of the anterior wall was significantly higher than that of the posterior wall ≥4 months after surgery (*p* < 0.001, *p* < 0.001, respectively).

All patients returned to previous levels of sports activity without symptoms and had not re-injured their ACL at final follow-up.

## Discussion

In this study, the SNR of the posterior wall was significantly higher than that of the anterior wall in each group with respect to the contrast enhancement on the wall of the femoral bone tunnel. Also, the SNR of the anterior wall was higher than that of the posterior wall in the groups after 4 months with respect to the tibial side. From these results, we showed that blood flow to the posterior wall of the femoral tunnel is richer than that to the anterior wall from the early postoperative stage, and that blood flow to the anterior wall of the tibial tunnel is maintained compared to the posterior wall 4 months postoperatively. We believe that our findings have revealed the advantageous area for the remodeling process. Especially on the femur side, the posterior area may be advantageous not only anatomically but also hemodynamically.

It is necessary to reconstruct the tendon graft biologically together with reacquisition of lower limb muscular strength, endurance, instantaneous power, and agility, as physical abilities, in order to enable a return to sports after ACL reconstruction surgery. Restoration of blood flow is important in the remodeling process of the tendon graft after ACL reconstruction surgery [21, 22]. Contrast MRA has been used as a method for evaluating blood flow to tendon grafts [23, 24]. However, the inflow route of blood was unknown, because contrast MRA images were obtained in the venous phase in which tendon grafts were sufficiently contrasted. In contrast, Arai et al. used MRA to visualize the blood flow to the bone tunnel wall and tendon grafts [17]. In addition, Terauchi et al. revealed that blood flow resumed from the middle genicular artery to the femur side and from the inferior genicular artery to the tibial side 2 months after surgery, indicating that these blood flows may play important roles in the remodeling process [18]. Furthermore, Kanamura et al. showed that blood flow reached the bone tunnel wall 2 months postoperatively and the graft tendon in the bone tunnel 3 months postoperatively, and that reconstruction was progressing 7 months after the surgery [19]. These results may reflect the blood flow reaching the posterior wall 2 months after ACL reconstruction and reaching the graft in the bone tunnel at 3 months postoperatively. Because angiogenesis from the bone tunnel wall to the graft tendon occurs specifically at 2–3 months postoperatively, we believe that athletic rehabilitation should be limited to linear exercise, avoiding rotational motion with a strong load on the graft tendons during that period.

**Fig. 5:**
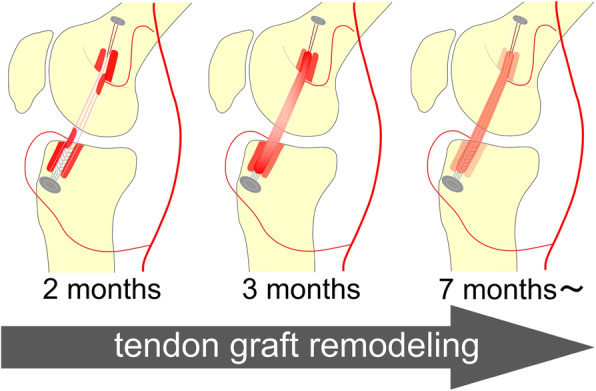
Hemodynamics of the bone tunnel wall and grafted tendon after ACL reconstruction are shown, including the results from this study and previous literature. Although blood flow reached the bone tunnel wall at 2 months after reconstruction from the middle genicular artery, blood flow resumes especially from the posterior bone tunnel wall on the femur side, which is closer to the artery. Blood flow was restarted within the tendon parenchyma at 3 months. On the tibial side, there was no significant difference in the early postoperative period, but finally blood flow was more abundant in the anterior bone tunnel wall closer to the inferior genicular artery, which is the feeding vessel of the tibial bone tunnel wall

The evaluation method using MRA could show the progression of remodeling by digitizing blood flow to the bone tunnel wall in vivo [19]*.* Blood flow toward the femur side resumes from the middle genicular artery, which is in the posterior of the knee joint, and toward the tibial side from the inferior genicular artery in the anterior. In the current study, contrast enhancement of femoral bone tunnel wall statistically differed between anterior and posterior from the early post-operative phase on the femoral side, but statistical difference on the tibial side was shown only 4 months after surgery. From these results, we considered that the blood flow is likely to resume from the inflowing artery to the bone tunnel wall near the blood vessel anatomically, and revascularization to the bone tunnel wall after ACL reconstruction may relate to the distance from the vessels, especially on the femoral side (Fig. 5). The difference in hemodynamics between the femur and tibia might affect the difference in the timing of blood flow resumption. Postoperative blood flow to the bone tunnel wall will be disadvantageous unless the location of the bone tunnel is anatomically appropriate. Therefore, it is desirable that the location of bone tunnel in the ACL reconstruction reproduces the anatomical attachment area from the viewpoint of the remodeling process. Elucidating the pathology by collecting the findings of the remodeling process after ACL reconstruction may develop more appropriate postoperative rehabilitation therapy.

This study has some limitations. First, we did not check the relationship of current results and biomechanical data. Second, a histological evaluation was not included. Third, the SNR of the bone tunnel wall is obtained from division of that of air, and the difference of these values themselves is meaningless. A fourth limitation is the time point of conducting MRA. We did not evaluate just after operation within 2 months. Also, not all patients were analyzed with contrast enhancement for 2 years. Fifth, postoperative management differed in some cases due to meniscal repair.

## Conclusions

In conclusion, we performed MRA after single-bundle ACL reconstruction using the semitendinosus tendon and quantitatively evaluated the blood flow toward the bone tunnel wall. This study showed that the blood flow after ACL reconstruction to the femoral bone tunnel is maintained from the posterior wall, and to the tibial side from the anterior wall 4 months postoperatively.

## Data Availability

Not applicable.
